# Prevalence of Intestinal Parasites in Captive Asian Elephants (*Elephas maximus* Linnaeus, 1758) in Central Nepal

**DOI:** 10.1002/vms3.70310

**Published:** 2025-03-27

**Authors:** Roshan Babu Adhikari, Madhuri Adhikari Dhakal, Purna Bahadur Ale, Ganga Ram Regmi, Tirth Raj Ghimire

**Affiliations:** ^1^ Nepalese Army Institute of Health Sciences (NAIHS) Kathmandu Nepal; ^2^ Alka Health Institute Pvt. Ltd. Lalitpur Nepal; ^3^ Third Pole Conservancy (TPC) Bhaktapur Nepal; ^4^ Department of Microbiology and Research and Development New Edge Microbials Albury Australia; ^5^ Department of Zoology Tri‐Chandra Multiple Campus Tribhuvan University Kathmandu Nepal; ^6^ Nepal Academy of Science and Technology (NAST), Khumaltar Lalitpur Nepal

**Keywords:** Asian elephants, captivity, endangered species, *Entamoeba*, polyparasitism, zoonosis

## Abstract

**Introduction:**

The Asian elephants (*Elephas maximus*), despite their larger physical structure and strength, are often attacked by microorganisms, like gastrointestinal (GI) parasites, resulting in higher morbidity and mortality.

**Aims:**

The current study aimed to determine the prevalence and diversity of GI parasites in the endangered Asiatic elephants reared in captivity in and around Chitwan National Park in Central Nepal.

**Methods:**

With age and sex variants, 63 fresh faecal samples (*N* = 63) were collected non‐invasively and transferred to the research laboratory for microscopic examination.

**Results:**

Our findings showed a 95.2% prevalence rate, along with 17 identified diverse species of GI parasites, including protozoa (6 spp.) and helminths (11 spp.) and two unknown species (1 protozoan and 1 helminth). The prevalence of protozoa (84.1%) was higher than that of helminths (77.8%). Female/cows and old‐age elephants were reported to harbour a higher rate of parasites. Sharing overlapping niches with domestic and wild animals, irregular medication and the existence of critical stressors were speculated to be the major risks for parasitosis.

**Conclusions:**

The captive elephant population in Central Nepal harbours a greater prevalence and huge diversity of GI parasites, most of which are implicated with serious pathological conditions and zoonotic potentiality. The presence of GI parasites must be considered a challenging threat. Thus, government bodies, non‐governmental organizations, elephant owners and conservationists need to participate in strategic medication and seek measures to lessen the probable health risk for sustainable conservation and welfare of the endangered species in Nepal.

## Graphical abstract

Gastrointestinal parasites (GIPs) are determining factors in the health of Asian Elephants. The current coproscopic study recorded 95.2% prevalence rates with 19 different GIPs from these animals. The major risk factors could be overlapping niches with domestic and wild animals, irregular medication and the existence of critical stressors.

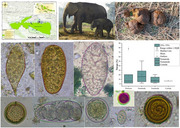

## 1 Introduction

The Asian elephant (*Elephas maximus* Linnaeus, 1758) (Family: Elephantidae, Taxonomic Serial Number: 584938, www.itis.gov) is the largest herbivorous mammal in Asia. It is one of the first creatures people have tamed since prehistoric periods (Csuti 2006). Geographically, their wild population has an uneven distribution and is highly restricted to the forest‐grassland ecotone (evergreen, semi‐evergreen, moist deciduous, and dry deciduous forests) of 13 countries in South Asia, including Nepal (Choudhury 1999; Olivier 1978). In Nepal, their wild population (estimated at 227 individuals) is distributed across Nepal's Terai Arc Landscape (TAL) (Ram and Acharya; WWF 2021), including the eastern lowlands of Terai and the Chure‐Siwalik area. However, their major home ranges include protected areas such as the Banke‐Bardiya Complex (*N* = 113), Chitwan‐Parsa Complex (*N* = 45) and Shuklaphanta Complex (*N* = 26) (Ram and Acharya 2020), with a total of 210 elephants in captivity, owned by both the government (*N* = 104) and private sectors (*N* = 106) (Ram and Acharya 2020). The government‐owned elephants have been used for emergency rescue and anti‐poaching missions, including patrolling, wildlife census and research. However, private‐owned elephants are for tourism business (Szydlowski 2020, 2022), like elephant safaris in and around the national park, and entertainment; for example, Nepal's most famous elephant polo, football and racing. Nevertheless, these sports were discontinued after 2018 due to criticism from animal rights activists (Prasain 2024).

Since the beginning of early civilization, Nepalese elephants have been used as vital tools of ceremonies, festivals, war and diplomacy (Ghimire et al. 2020; Mocko and Barnhart 2018; Szydlowski 2020). However, poaching, habitat loss, habitat fragmentation, human‐elephant conflict and mistreatment in captivity have been described as the significant factors for the decline of these megaherbivores (Ghosh 2021; Qiu 2022). In addition, intimidating factors like natural disasters, diseases (mainly tuberculosis), foot problems, indigestion, endotheliotropic herpes virus‐hemorrhagic disease (EEHV‐HD), gastrointestinal (GI) parasitism, physical injuries, including other diseases, and more recently, political strife (Boarder closed) and anthropause (Gautum and Koju 2022; Paudel et al. 2014; Szydlowski 2022) have been implicated to directly impact the survival, embellishments and welfare of the captive elephant population. As a result, they are categorized as “endangered” species under the IUCN Red List and listed in CITES Appendix I (IUCN 2020). Among many endangerment threats, GI diseases can be critical for their health, well‐being and survival. Moreover, via enhanced competition, parasites modify the evolution and ecology of every interaction, leading to species extinction (Hamilton and Zuk 1982; Price et al. 1986; Yan 1996), suggesting parasitic faunae are important in elephants' survival. Their population is relatively stable in contrast to those of one‐horned rhinos and Bengal tigers within the country's TAL despite the continuous conservation attempts and the execution of essential action plans (GoN 2015), indicating the existence of one or more threats for these mammals. Based on previous reports of parasite‐mediated hyperalbuminemia, anaemia, gastroenteritis, emaciation, diarrhoea, reduced appetite and deaths of both African and Asian elephants (Fowler and Mikota 2006; Obanda et al. 2011; Perera et al. 2017; Vitovec et al. 1984), GI parasites should be recognized an important part of risks in elephants. Although numerous studies in Asian elephants have reported varied diversities of protozoa and helminths, with prevalence rates ranging from 17.2% to 100% (Abeysekara et al. 2018; Abhijith et al. 2018; Chel et al. 2020; Saseendran et al. 2004), such studies in Nepal are very limited. Existing studies have detected the eggs of trematodes such as *Dicrocoelium* sp., *Fasciola* spp., *Paramphistomum* sp., *Schistosoma* sp., cestodes like *Anoplocephala* sp., *Moniezia* sp. and nematodes like Strongyle, *Strongyloides* sp. and *Trichostrongylus* sp. in the dung samples (Dahal et al. 2023; Karki and Manandhar 2007; Pandit et al. 2015; Shahi and Gairhe 2019). However, the reports of protozoan diversity, associated disease epidemiology and potential zoonosis are yet to be thoroughly evaluated and explained. Therefore, the current study was designed to assess the prevalence and diversity of GI protozoa and helminths and identify the zoonotically significant parasites associated with domestic and captive Asian elephants in Central Nepal.

## 2 Materials and Methods

### 2.1 Study Area

**FIGURE 1:**
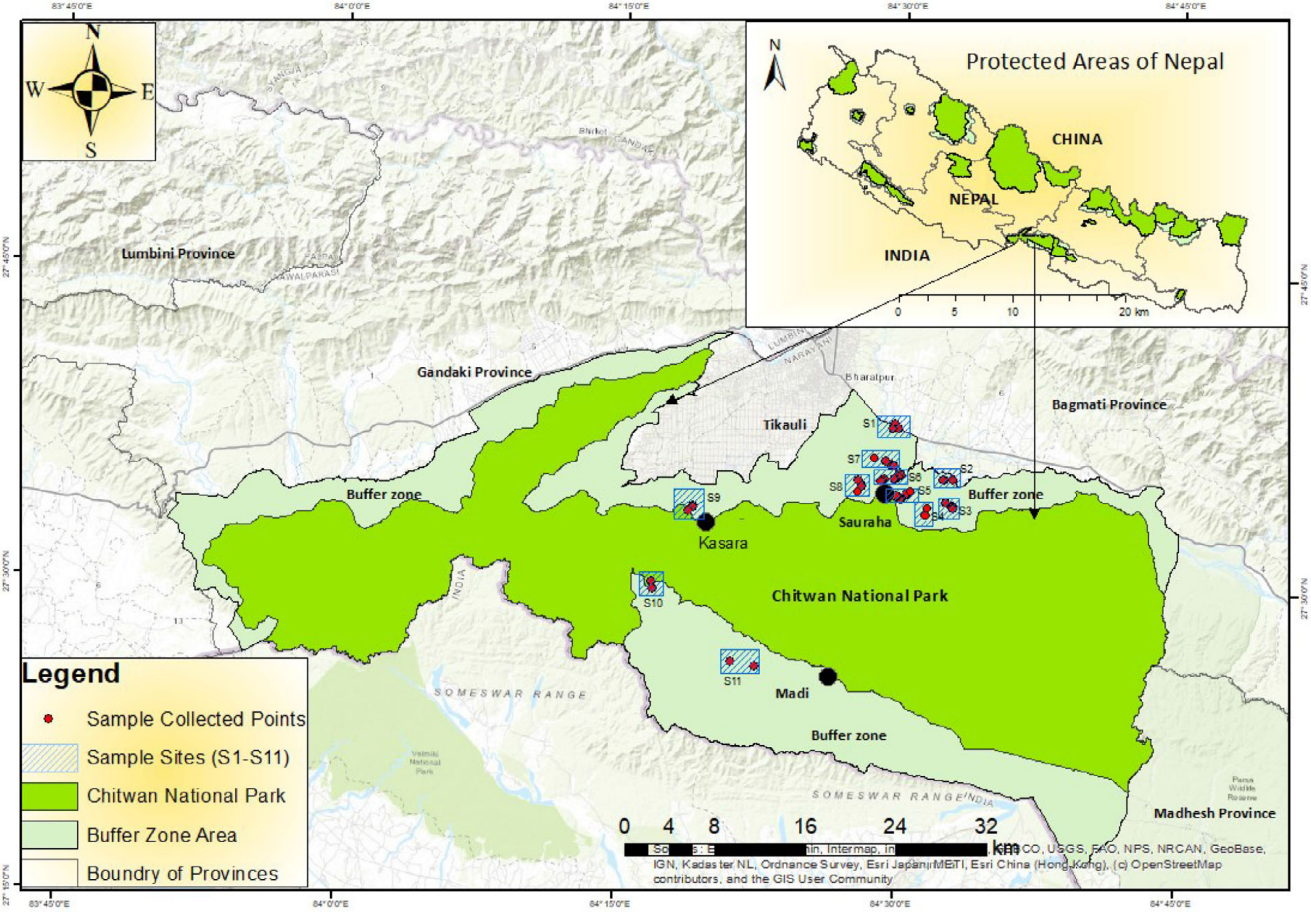
Map of study area.

The current study area included the adjoining periphery of Chitwan National Park (CNP), the oldest national park in Nepal listed in the World Heritage Site in the Chitwan district in South‐Central Nepal (Figure 1). The area falls under the subtropical to tropical lowlands of Nepal's TAL and has a tropical monsoon climate, with high humidity all around the year. It has mainly three seasonal variations: Summer: March to June, Monsoon: May to September and Winter: October to February (https://www.junglesafariresort.com/climate.php). It receives an average annual maximum temperature (21°C–35°C), an average minimum temperature (10°C–24°C) and an average annual precipitation (5–530 mm) (R. B. Adhikari, Adhikari Dhakal, Ale, et al. 2023). The major vegetation includes tropical sal forest (*Shorea robusta*), riverine forest, khair (*Acacia catechu*), sissoo (*Dalbergia sissoo*), simal (*Bombax ceiba*) and smaller to taller grasses, including the elephant grass (*Saccharum* sp.), *Phragmites karka* and *Arundo donax* (NTNC‐BCC & CNP, 2020). Currently, the Chitwan‐Parsa Complex supports 45 wild and 97 captive elephants around the periphery of CNP (mainly Sauraha) (Gautum and Koju 2022). The number of captive elephants is owned by the government (*n* = 31), the National Trust for Nature Conservation (*n* = 6) or the private sector (*n* = 60) (Gautum and Koju 2022). Besides, the area also includes the Elephant Breeding Center, the only centre for ex‐situ conservation that promotes scientific research and gene pool conservation through a captive breeding program, and the Elephant Bathing Center at Sauraha and Meghauli, where tourists enjoy the great experience of bathing with the elephants. Furthermore, the study areas also include the adjacent territories of Twenty Thousand Lake, Kumal Lake, Tikauli Lake and Rhino Lake, the river basins of Budi Rapti, Rapti, Khageri, Reu, Panchanadi and others along their branching tributaries in Sauraha and Madi areas. The area primarily serves as an ecotone region, where humans, including domestic animals and wild animals, like elephants, one‐horned rhinos and deer co‐exist, sharing the available resources.

### 2.2 Faecal Sample Collection, Preservation and Transportation

The faecal samples were collected from November 2020 to March 2021 from the preferable resting sites and government and private elephant sheds, and 63 faecal samples (Males/Bulls: 5, Females/Cows: 58) were opportunistically and non‐invasively collected. Information regarding age, sex, working hours, disease condition and deworming history was collected through a structured questionnaire survey with the associated caretaker (Mahout). The total elephant population in the study was divided into three categories based on their age: Young (<16 years; 9 individuals), Adults/Working (16–70 years; 48 individuals) and Old/Retired (>70 years; 6 individuals). The fresh dung sample, immediately after defecation, was only collected for the laboratory investigation. The piles of dung samples (*Thaso in Nepali*) were initially macroscopically examined and photographed for faecal consistency and then for the presence or absence of blood, mucus and detached gravid proglottids. Using gloved hands, a metallic spatula and forceps were employed to pick up the required amount of faecal samples from different portions of the pile into a 50‐millilitre (mL) sterile vial. These samples were immediately preserved in 2.5% weight/volume (w/v) potassium dichromate solution (K_2_Cr_2_O_7_), and then transported to the laboratory for microscopic examination and other necessary investigations.

### 2.3 Laboratory Processing and Examination

For laboratory analysis, four different techniques, like direct wet faecal mount (DWM), faecal concentration (sedimentation and flotation techniques) and acid‐fast staining techniques, were used based on the standard protocol previously designed (J. N. Adhikari et al. 2021; R. B. Adhikari et al. 2020; R. B. Adhikari, Adhikari Dhakal,  et al. 2022; R. B. Adhikari, Ghimire, et al. 2025; Sapkota et al. 2020). About ten grams (g) of the faecal sample were taken from the vials into a beaker, and 15 mL of normal saline (0.9% w/v NaCl) was added. The mixture was stirred vigorously, filtered using a metallic tea strainer into a Petri dish, and finally, the filtrate was used for microscopic examination.

#### 2.3.1 Direct Wet Faecal Mount

After careful stirring, a single drop of faecal filtrate was kept on the glass slide with or without Gram's iodine stain using a plastic dropper. The faecal smear was then covered with a coverslip and examined under the microscope (10× and 40×).

#### 2.3.2 Saturated Salt Flotation Technique

Initially, 2–3 mL of the faecal filtrate and 12 mL of normal (0.85% w/v) saline (NaCl) were poured into a 15 mL conical centrifuge tube and centrifuged (1200 revolutions per minute, rpm × 5 min). The supernatant was discarded, and 12 mL of flotation media (45% w/v NaCl) was added to the tube for subsequent centrifugation (1200 rpm × 5 mins). Then, without discarding the supernatant, the flotation media was added drop by drop to fill it. After that, a coverslip was placed at the top of the tube so the flotation media touched it and left it undisturbed for about 10 min. Finally, the coverslip was removed, placed on a glass slide and examined under the microscope (10× and 40×) with or without Gram's and Lugol's iodine.

#### 2.3.3 Formalin‐ethyl Acetate (FEA) Sedimentation

Ten mL of 10% formalin and 4 mL of ethyl acetate were added to the faecal sediment obtained after first centrifugation and then re‐centrifuged (1200 rpm × 5 min). Then, the supernatant was discarded, and a single drop of sediment was used for microscopic examination (10× and 40×).

#### 2.3.4 Acid‐Fast Staining (AFS)

Thin smears were prepared using the sediment obtained after FEA sedimentation. Initially, the air‐dried smears were fixed in absolute methanol for 2 min and then flooded with carbol fuchsin for about 15 min. The smears were then destained with distilled water and acid alcohol and then counterstained with malachite green for a minute. Finally, following a gentle wash with cool water, the smears were allowed to dry at room temperature and inspected under a microscope using immersion oil (100×).

#### 2.3.5 Estimation of Parasitic Burden/Severity of Infection

A ‘2Cell McMaster Counting Slide’ (Hawksley and Sons Ltd.) was employed to quantify the burden of parasitic infection. It was estimated by counting the number of eggs and oocysts released in each gram of the faecal sample. The procedure was entirely based on the instructions provided by the manufacturer company and literature previously explained (R. B. Adhikari et al. 2023).

### 2.4 Parasite Identification

Photomicrography of all the reported parasitic bodies, including cysts, oocysts, trophozoites, eggs and larvae, were captured with a camera (SXView 2.2.0.172 Beta (Nov 6, 2014) Copyright (C) 2013–2014) attached with the microscope (Optika Microscopes Italy, B‐383PLi). Then, ImageJ 1.51 k (National Institute of Health, USA) software was used for morphometric analysis. The identification of parasites was based on their morphological characters previously explained in the literature (Soulsby 2012; Zajac et al. 2021). Furthermore, *Fasciola* sp. and *Paramphistomum* sp. were confirmed based on their staining property with methylene blue (R. B. Adhikari et al. 2022; Hansen and Perry 1994). Operculated large‐sized eggs with a golden yellow colour with methylene blue were considered *Fasciola* spp., while those remaining unstained/colourless were considered *Paramphistomum* sp.

### 2.5 Data Analysis

All the parasitological data generated were encrypted and entered into Microsoft Excel 2016 and OriginPro 2024 (Learning Edition) spreadsheets. To calculate the percentage prevalence of each parasite, the number of positive samples was divided by the total number of samples in the population and then multiplied by 100. The data were analysed using GraphPad Prism 2007 and OriginPro 2024 and applied Pearson's Chi‐squared (χ^2^) tests and Fisher Exact test to determine statistically significant differences at a 95% confidence interval (CI) (*p* < 0.05).

## 3 Results

**FIGURE 3:**
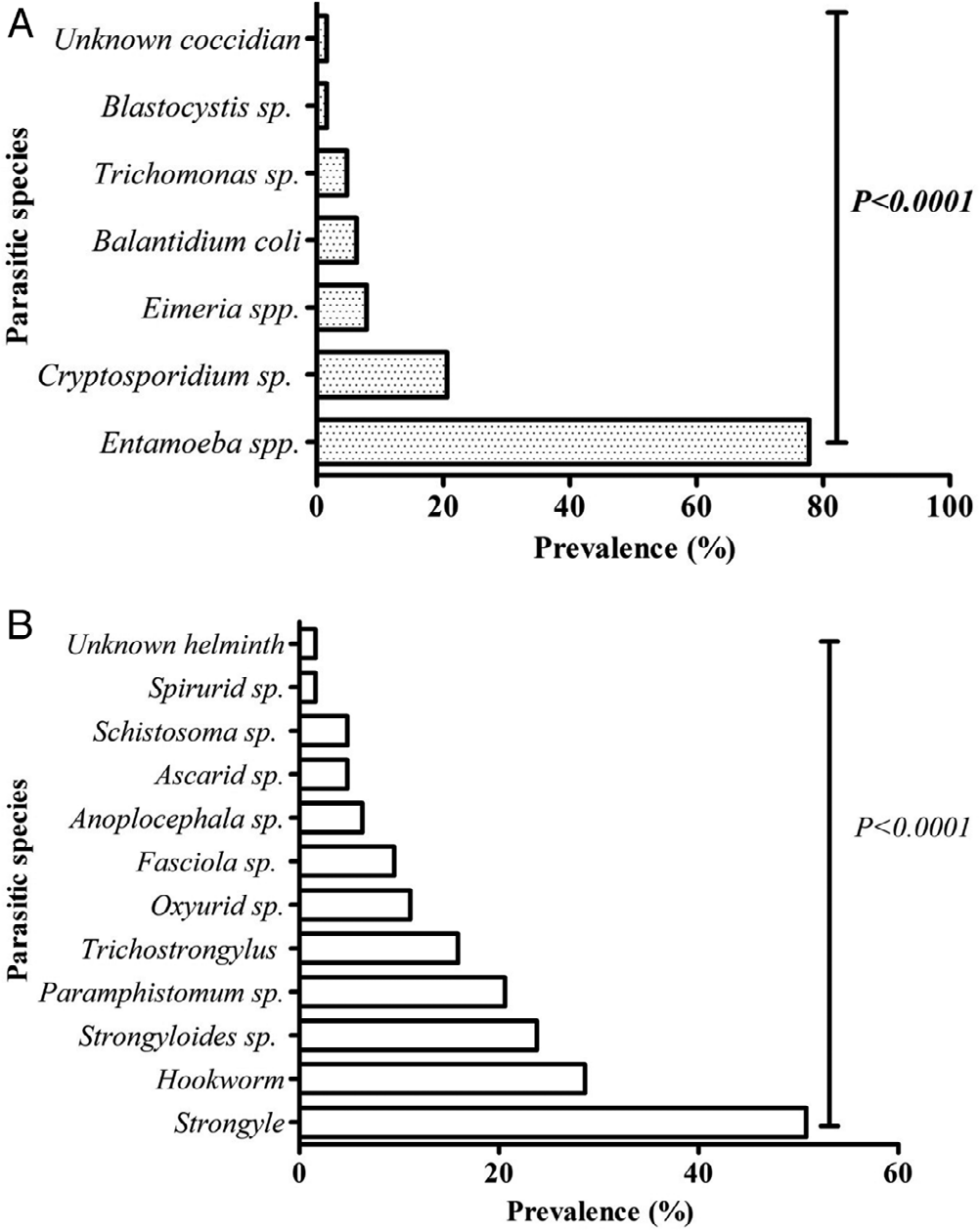
Prevalence of intestinal parasites in Asian elephants. (A) Prevalence of protozoa. (B) Prevalence of helminths.

**FIGURE 4:**
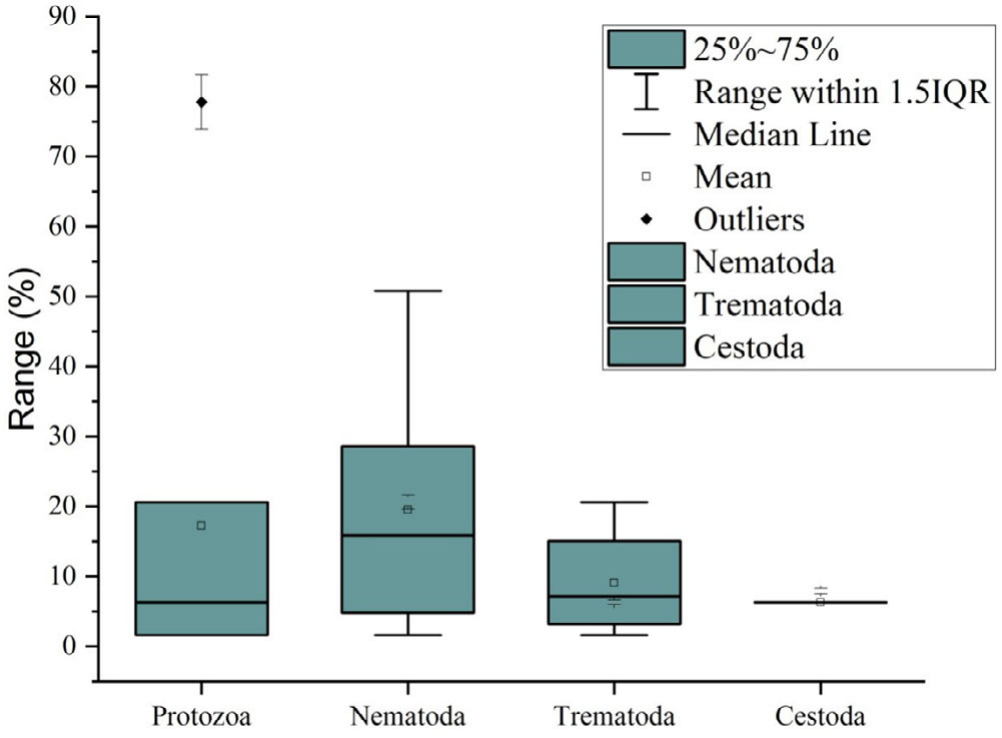
Ranges of prevalence rates of different parasite groups in Asian elephants.

In the present study, coproscopy revealed that 95.2% (60/63) of the faecal samples obtained from captive elephants were positive for at least one intestinal parasite species. The sensitivity of three laboratory techniques yielded distinct results while detecting GI parasites. For instance, the DWM, FEA and floatation techniques revealed 76.7% (46/60) (14 species), 100% (60/60) (17 species) and 86.7% (52/60) (11 species) of positive samples, respectively. However, 19 overall diverse species of parasites (17 known and two unknown) were detected in this study. Taxonomically, protozoa included Sarcodina (*Entamoeba* spp., *Blastocystis* sp.), Ciliata (*Balantidium coli*), Flagellata (*Trichomonas* sp.) and Coccidia (*Cryptosporidium* sp., and *Eimeria* spp.) with different prevalence rates (*p* < 0.0001). Similarly, helminths included Trematoda (*Fasciola* spp*., Paramphistomum* spp., *Schistosoma* sp.), Cestoda (*Anoplocephala* sp.) and Nematoda (Ascarid, sp., hookworm, Strongyle, *Trichostrongylus, Strongyloides* sp., Spirurid sp. and Oxyurid sp.) with different prevalence rates (*p* < 0.0001). *Entamoeba* spp. and *Cryptosporidium* sp. were the most frequently recorded protozoa, while Strongyles and hookworm were the most frequently recorded helminths (Figures 2 and 3). Furthermore, the prevalence of overall protozoan parasites was higher than the helminths (84.1% vs. 77.8%), and the occurrence of nematode parasites (78.2%) was higher than trematodes (25.4%) and cestodes (6.3%) (Figure 3, 4). Interestingly, only a sample (1.7%) was positive with protozoan species alone, while 23 (38.3%) were positive with the helminths, and 36 (60%) were co‐infected with both protozoan and helminth parasites (Figure 3).

FIGURE 2A Gastrointestinal protozoan parasites identified in Asian elephants (Microscopic photographs under 40x, and 100× magnification of objective lens of microscope). (A) Cyst of *Entamoeba* sp.1 (10×10 µm) after FEA sedimentation technique. (B) Cyst of *Entamoeba* sp. 2. (17×16 µm) after DWM technique. (C) Cyst of *Entamoeba* sp. 3. (23×23 µm) after DWM technique. (D) Cyst of *Blastocystis* sp. (16×16 µm) after DWM technique. (E) Oocyst of unknown coccidian (27×25 µm) after flotation technique. (F) Oocyst of *Cryptosporidium* sp. (6×6 µm) after AFS technique. (G) Oocyst of *Eimeria* sp. 1 (27×17 µm) after DWM technique. (H) Cyst of *Balantidium coli* (51× 49 µm) after FEA sedimentation technique. B Gastrointestinal helminth parasites identified in Asian elephants (Microscopic photographs under 40× magnification of objective lens of microscope). (A) Egg of Spirurid sp. (51×17 µm) after flotation technique. (B) Egg of hookworm (61×35 µm) after flotation technique. (C) Egg of Ascarid sp. (46×44 µm) after FEA sedimentation technique. (D) Egg of *Anoplocephala* sp. (57×57 µm) after flotation technique. (E) Egg of Oxyurid sp. (44×26 µm) after flotation technique. (F) Egg of *Strongyloides* sp. (64×40 µm) after flotation technique (G) Egg of Strongyle 1. (74×49 µm) after flotation technique. (H) Egg of unknown helminth (67×46 µm) after FEA sedimentation technique. (I) Eggs of Strongyle 2. (79×40 µm) after flotation technique. (J) Egg of Strongyle 3. (87×50) after DWM technique. (K) Egg of Strongyle 4. (93×44 µm), after flotation technique. (L) Egg of Strongyle 5. (114×56 µm) after flotation technique. (M) Egg of *Schistosoma* sp. (128×48 µm) after FEA sedimentation technique. (N) Egg of *Fasciola* sp. (112×58 µm) after FEA sedimentation technique. (O) Egg of *Paramphistomum* sp. (131×66 µm) after FEA sedimentation technique.

**Figure:**
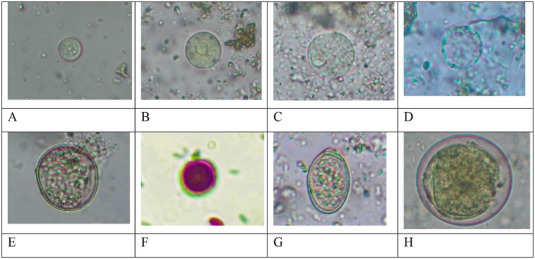

**Figure:**
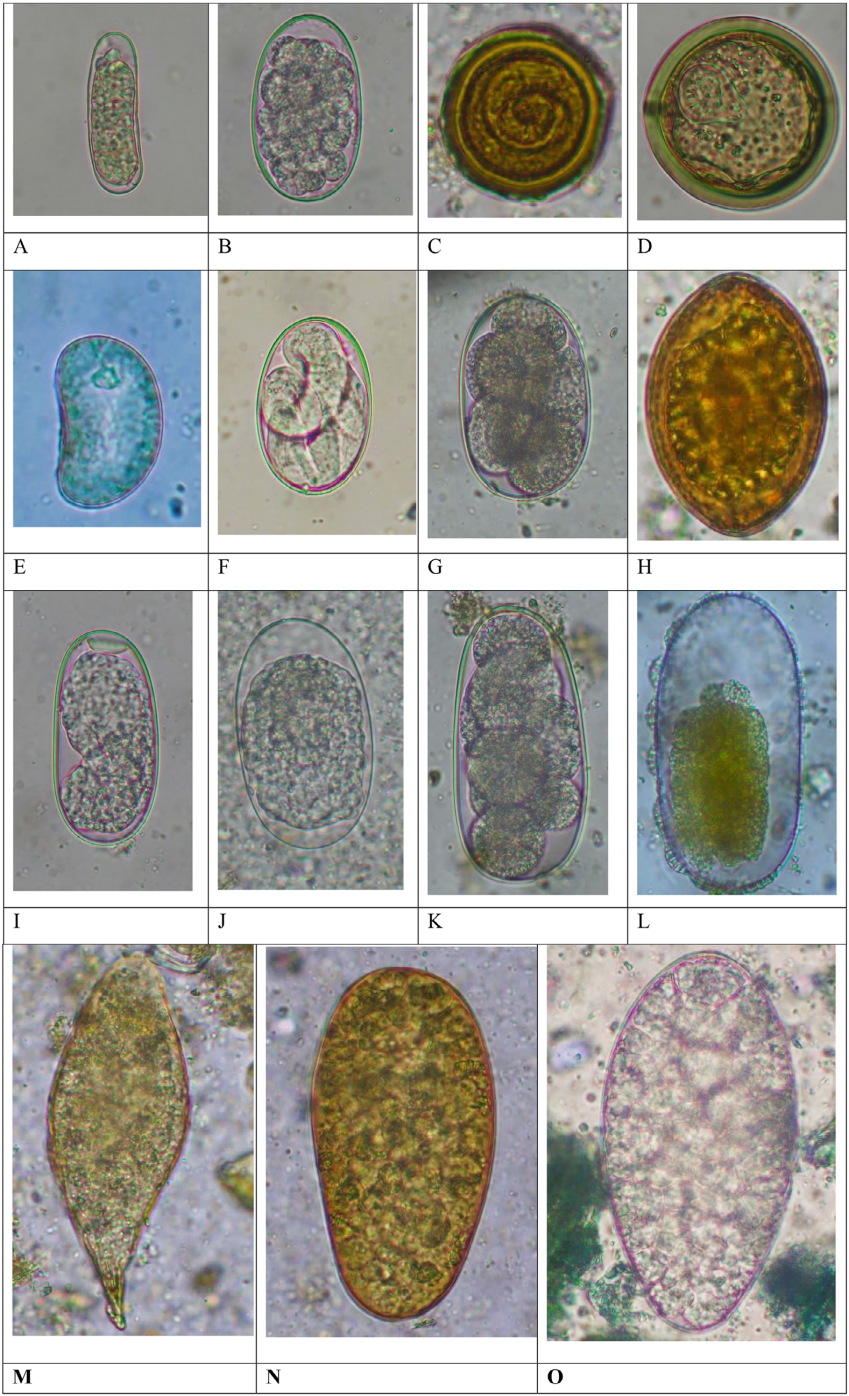

Remarkably, the mixed pattern of infection was higher than mono parasitism. Triple infection (23.8%) was the highest, followed by double (19%) and septuple infection (4.8%) was the least.

Considering the sex and age variant, the prevalence of GI parasites was higher in females/cows (96%) than in male/bull elephants (80%), with significant differences in their prevalence rate (Fisher Exact, *p* = 0.0008). Similarly, old/retired elephants showed a higher (100%) prevalence rate than adults (97.9%) and young (77%), and this difference in the prevalence rate was statistically insignificant (Chi‐square, *p* < 0.0001).

**FIGURE 5:**
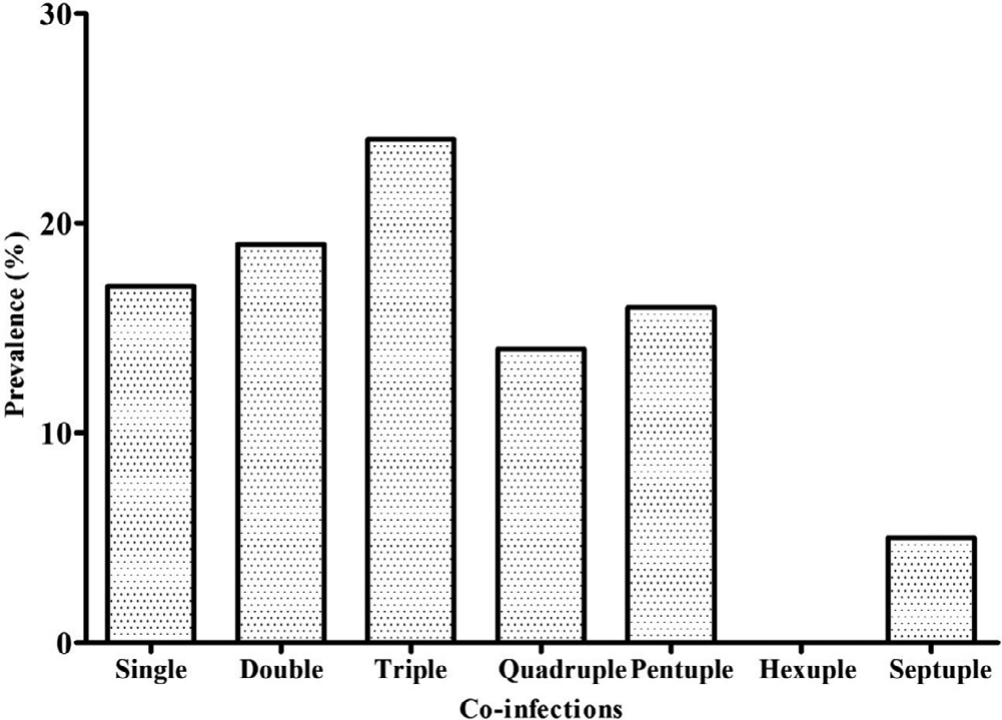
Prevalence (%) of single and mixed infection of GI parasites in Asian elephants.

Interestingly, we also recorded four morphotypes of *Eimeria* spp. (Size: 23–35×16–24 µm), two morphotypes of *Fasciola* spp. (Size: 112–142×58–68 µm), two morphotypes of *Paramphistomum* spp. (Size: 115–144×65–70 µm), and five morphotypes of strongyle eggs (Size: 74–114×40‐56 µm) (Figures 2 and 5).

Furthermore, the burden was estimated on the random selection of 10% faecal samples positive for particular parasitic species. For example, the parasitic burden as generated by McMaster (epg/opg) count was *Eimeria* spp. (Mean OPG: 100–1200, *n* = 4), hookworms (Mean EPG: 100–2300, *n* = 10), Strongyles (Mean EPG: 100–2600, *n* = 15), *Strongyloides* sp. (Mean EPG: 100–1300, *n* = 5), 100–200 eggs of Spirurid sp. (Mean EPG: 100–200, *n* = 1) and Oxyurid sp. (Mean EPG: 100–600, *n* = 2).

## 4 Discussion

The coprological investigation in the present study revealed a 95.2% (60/63) prevalence rate of GI parasites in the captive elephant population in Central Nepal. This outcome is slightly lower than the earlier reports from the captive elephant population in Nepal (100%; *n* = 20) (Karki and Manandhar 2007) and Myanmar (100%; *n* = 10) (Chel et al. 2020) and the free‐ranging wild population in India (100%; *n* = 50) (Vimalraj and Jayathangaraj 2015). On the other hand, this result is slightly higher than the outcomes from the captive elephant population in India (17.2% ‐ 74.5%; *n* = 55 ‐ 99) (Abhijith et al. 2018; Saseendran et al. 2004; Vanitha et al. 2011), Sri Lanka (25%; *n* = 20) (Abeysekara et al. 2018) and Nepal (47.6%; *n* = 103) (Dahal et al. 2023) as well as the wild elephant population in India (71%–93.3%; *n* = 25‐320) (Arunachalam et al. 2007; Suresh et al. 2001; Vidya and Sukumar 2002), Sri Lanka (71%; *n* = 7 to 93.3%; *n* = 45) (Abeysekara et al. 2018; Hewavithana et al. 2022) and Nepal (95%; *n* = 40) (Shahi and Gairhe 2019). The inequality in the prevalence rate of parasites in these studies might have occurred due to the methodological contrast and variation in three different factors, often considered a disease triangle (Ghimire, Regmi, et al. 2020; Scholthof 2007). This includes host factors (age, sex, behaviour, immune status and others), environment (climatic condition, altitude, geography and others) and the nature of infecting parasites (virulence, pathogenicity and others). However, we speculate three significant factors that might have enhanced the prevalence and diversity of GI parasites.

The first factor is attributed to the sharing of overlapping niches by domestic and wild animals. Since most pathogens, including parasites, infect multiple hosts (Woolhouse et al. 2001), there is potential for cross‐transmission within the individual of the same species or different in a multi‐host and multi‐parasite ecosystem (Walker et al. 2017). In the study area, buffer zone areas, community forest pasturelands, water holes and wetlands are commonly shared by wild and captive elephants and other wild herbivores. In this context, captive elephants could have acquired parasites in the ecotones through various sources, such as faecally contaminated soil, water bodies and even mechanical and biological vectors. Although livestock grazing is entirely restricted inside the CNP, it can be observed outside, serving as the main route of parasitic transmission for elephants. Notably, domestic animals, like buffaloes and horses near the study area sharing the pasturelands, were diagnosed with a few parasites with cross‐transmission potentialities, like *Cryptosporidium*, *Balantidium coli, Paramphistomum*, *Fasciola*, *Trichostrongylus* and other nematodes (R.B Adhikari et al. 2024; R. B. Adhikari, Dhakal, et al., 2022).

The second factor in our observation is an irregular pattern of deworming that existed more commonly in private elephants. This problem in captive elephants was more pronounced during the COVID‐19 pandemic wave and, thereafter, the discontinuation of national and international tourism. During that period, due to a sudden fall in the safari business, elephants faced little care for veterinary health and parasitic treatments, including dietary distress (Szydlowski 2022). Even though a few private elephant owners and mahouts of government elephants claimed the continuation of regular deworming, it lacked provisions for anti‐protozoal drugs. This could be due to the reason for a higher prevalence of protozoan parasites than those of helminths in the present study. Physical and dietary stress upregulates glucocorticoid hormone that can downregulate immune protection against parasitism (Martin 2009; Romeo et al. 2020). For example, vulnerability to nematode infection in elephants due to the deficiency of protein and energy as dietary components has been explained (Chapman et al. 2005). This hypothesis might be true because of the predominance of nematodes (76.2%) in the current observations of elephants. Similarly, dietary stress and parasitism synergistically resulted in mass mortalities in African elephants in Kenya (Obanda et al. 2011). In addition, repeated exposure to harsh training, including physical and mental control management practices and inappropriate resting stables, for example, in the current elephant populations (Kurt 2005; Szydlowski 2022; Vries 2014), might have enhanced parasite acquisition. However, nutritional status, stress, management practices, and parasitism studies should be further conducted to verify this opinion.

The finding regarding the predominance of protozoan species (84.1%) over that of helminths (77.8%) is on par with the result obtained from the captive elephant population in Sri Lanka (Abeysekara et al. 2018). However, contrasting results were obtained among wild populations (Abeysekara et al. 2018) in the same geography. The reason could be the traditional practice of medication, which primarily excludes anti‐protozoal treatment similar to ours. Although *Entamoeba* spp. with three cyst morphotypes (77.8%) are prevalent in current elephants, the finding from Sri Lanka was lower (10%–22.2%) among captive, semi‐captive and wild populations (Abeysekara et al. 2018). Different species of *Entamoeba* have been described around the globe, for example, *Entamoeba* RL10/*E. hartmanni* in an Asian elephant at Amsterdam Zoo (Jacob et al. 2016), *Entamoeba moshkovskii* in African bush elephants in Namibia (Parfrey et al. 2014) and *Entamoeba* sp. in Sri Lanka under similar settings (Aviruppola et al. 2016). Notably, both of these species can potentially transmit diseases to humans, indicating the need for a molecular study for species identification and evaluating the risk of zoonotic transmission in the current elephants. Similar to the *Cryptosporidium* sp. in the current dung samples, its oocysts have also been detected in captive African elephants at the Barcelona and the Lisbon Zoo (Gracenea et al. 2002; Silva et al. 2022). Its prevalence rate (20.6%) is slightly lower than that of wild African elephants in South Africa (25.8%) (Samra et al. 2011). These coccidia generally infects the immunocompromised host, implying that young and ageing elephants are always at greater infection risk.

The current detection rate of *Eimeria* spp. (7.9%) is lower than reported in Indonesia (25%) (Melia et al. 2020) and Nigeria (7.8%–30.23%) (Mbaya et al. 2013) in the Sumatra and African elephants, respectively. The presence of *Eimeria bovis* in the elephant has been claimed (Mbaya et al. 2013). Therefore, a molecular study is needed for species identification and to verify whether buffalo‐specific *Eimeria* spp. are circulating in the study area (Aryal et al. 2022) infects the elephants. Similarly, the currently reported *Balantidium coli*, a zoonotic ciliate, has also been confirmed in a pathologically ill female Asian elephant that suffered from mucoid watery diarrhoea, appetence, lethargy, weight loss and bilateral lacrimation (Thakur et al. 2019). This ciliate is highly prevalent in livestock, such as buffaloes (R. B. Adhikari, Adhikari Dhakal, et al. 2022; R. B. Adhikari and Ghimire 2021), pigs (R. B. Adhikari, Adhikari Dhakal, et al. 2021), horses (R.B. Adhikari et al. 2024), wild herbivores, like deer (Baral et al. 2019), including humans (R. B. Adhikari, Parajuli, et al. 2021) in the surrounding periphery, indicating faecal samples outside national park might be important reservoirs of zoonosis to local people. Uniquely, *Blastocystis* sp. has also been identified in a dung sample. Previously, *Blastocystis* subtype 11 (ST 11, 96.4% homology to zoonotic strain ST 1) was found in captive elephants at a zoo in Bangladesh (Li et al. 2019); however, the occurrence of diverse subtypes remains unclear due to lack of further confirmation.

Strongyle (50.8%) was the highly reported species considering the helminths. This group of nematodes has been recorded from a wild population in Sri Lanka (100%) (Abeysinghe et al. 2017), captive elephants in Nepal (10.1%–90.4%) (Dahal et al. 2023), India (37%–58.1%) (Abhijith et al. 2018; Vanitha et al. 2011) and Sri Lanka (90.4%) (Abeysinghe et al. 2017). Strongyles in elephants are likely similar to those of domestic livestock (Fowler and Mikota 2006), suggesting the possibility of cross‐transmission among livestock and elephants upon grazing on the common pasturelands. Another nematode reported was hookworm, which had a 28.6% prevalence rate lower than that reported in India (1.82%) (Abhijith et al. 2018). Similarly, many adult bile duct hookworms (*Grammocephalus varedatus*) were reported in a post‐mortem examination of wild elephants in the same geography (Kumar et al. 2011). Pathologically, elephants suffer from anaemia, weakness, hepatic insufficiency, the proliferation of lymphoid tissue, ulcers and necrotic foci in their bile duct (Chandrasekharan et al. 1995; Fowler and Mikota 2006), indicating hookworm is a critical parasite in these mammals.

Similarly, *Strongyloides* sp. (23.8%) was on par with the result of captive populations in Nepal (23%) (Pandit et al. 2015) but slightly higher than the record of a wild population in India (16%) (Vimalraj and Jayathangaraj 2015). All these nematodes infect a wide range of vertebrates, including humans. They can be increased in elephants due to their playing and swimming behaviours, including throwing dirt on their backs, which possibly protects them from sun and arthropod bites. In addition, 4.8% of the elephants were infected with the *Ascarid* sp. Since eggs of this nematode get embryonated in soil, consuming contaminated feed and fodders and the soil‐eating habit of elephants (Houston et al. 2001) might contribute to significant risk.

Regarding cestodes, the presence of *Anoplocephala* sp. (28.6%) was slightly lower than reported from wild populations in India (46%) (Vimalraj and Jayathangaraj 2015). Soil‐dwelling minuscule arachnids called oribatid mites act as the intermediate hosts of the tapeworm; thus, elephants acquire it upon grazing and consuming soil‐contaminated fodder. Interestingly, five mite species (*Galumnaflabellifera orientalis, Scheloribates latipes, S. praeincisus, Protoribatesseminudus* and *Poneracantha triangularis*) collected from the dung piles near the bedding of captive elephants in India were positive with the infective cysticercoids (Michael McAloon 2004). Therefore, the deposition of dung piles near the stable and the provision of fodder on the ground might be risk factors for cestodiasis. In addition, elephants with GI infections usually develop a habit of mud‐eating (Leung 2017). Even if it could be a way of self‐medication (Houston et al. 2001), it favours the larval invasion upon the ingestion of soil mites.

Trematodes are critical in elephants from around the globe. For example, *Paramphistomum* sp. (20.6%) was lower than previous reports on the wild population in Nepal (28.95%) (Shahi and Gairhe 2019) and higher than on the captive population in India (14.29%) (Chichilichi et al. 2019). Another digenetic trematode, *Fasciola* sp. (9.5%), was higher than the previous report in Nepalese captive elephants (9%) (Pandit et al. 2015) but lower than the report in a native wild population (39.47%) (Shahi and Gairhe 2019) and Malaysia (70.2%) (Hing et al. 2013). Fasciolids, like *Protofasciola robusta*, *Fasciola jacksoni* and *Fascioloides magna* can lead to serious pathology and deaths of elephants (Rajapakse et al. 2019; Singh et al. 1994; Vitovec et al. 1984). Similarly, *Schistosoma* sp. had a prevalence rate of 4.8% which was much lower than the local reports in captive (90%) (Karki and Manandhar 2007) and wild populations (71.05%) (Shahi and Gairhe 2019). This fluke can lead to anaemia, poor growth and digestive and reproductive stress in domestic herbivores (Dargie 1980). It was also found in India (9.1%) (Arunachalam et al. 2007) and the Central African Republic in African elephants (Brant et al. 2013). Considering the Elephant schistosome, *Bivitellobilharzia nairi*, it also infects the greater one‐horned rhinoceros (Devkota et al. 2014), suggesting the possibility of cross‐transmission among the megaherbivores in CNP. Since the infective tailed‐cercariae larvae remain free, swimming in freshwater bodies, wallowing, swimming and drinking activities in contaminated water contribute to schistosomiasis.

Risk factors of such trematodes include freshwater snails, *Planorbis* and *Lymnaea*. Interestingly, snail hosts, like *Bellamya bengalensis*, *Gabbia orcula*, *Gyraulus euphraticus*, *Indoplanorbis exustus*, *Lymnaea luteola*, *Melanoides tuberculata*, *Pila globosa*, *Thiara granifer* *a* and *Thiara lineata*, have been identified carrying infective cercariae of trematodes within the periphery of CNP (Devkota et al. 2011). Similarly, the presence of aquatic plants (*Trapa* spp./*Eichhornia* spp.) and the usual behaviour of elephants, like swimming and wallowing or consumption of water containing the cercaria larvae, remain significant risk factors (Horak 1971; Nelwan 2019).

Sex‐wise parasite infection was notable, and it was higher in females, similar to findings from Nepal (Dahal et al. 2023) and India (Abhijith et al. 2018). Since all the working elephants in the study area were females and remained frequently interactive with the herd, greater acquisition of parasites by females was possible. In contrast, males were solitary and primarily used for breeding. Unfortunately, we could not address more than two adult males, as working elephants are mainly female in the study sites. In addition, old/retired elephants showed a higher prevalence (100%) of parasites than adults and young. By nature, ageing downregulates the immune system (Montecino‐Rodriguez et al. 2013) which is why parasitic infection, including the infection by opportunistic parasites like *Cryptosporidium*, *Entamoeba* and *Strongyloides*, was predominantly detected in old elephants. Moreover, this particular age group of animals faced the worst dietary and care stress brought on by the COVID‐19 pandemic and subsequent lockdowns.

Notably, mixed infection was more prevalent than mono parasitism. Polyparasitism, either protozoa‐protozoa, protozoa‐helminths or helminths‐helminths species are evidenced to synergistically exacerbate pathology in naturally infected domestic (R. B. Adhikari and Ghimire 2021) and wild animals (Lello et al. 2005), including the experimental models (Graham et al. 2005). The synergistic effect of polyparasitism and food starvation might be the reason behind the mass mortality of elephants in Kenya (Obanda et al. 2011), indicating limited nutrition supply and mixed infection are problematic for their health. Nevertheless, the possibility of negative interaction between the co‐infecting parasites also exists (Schjørring and Koella 2003). In the current study, 60% of elephants were found to harbour multiple protozoa and helminths at a time, leading to moderate to heavy parasitic burden as estimated by epg/opg. Theoretically, this could result in virulence and infection severity, although it is practically unclear how the parasitic interaction affects the disease outcome. Synergistic pathology occurs not only among parasites but also among various groups such as bacteria and viruses or fungi (Jolles et al. 2008; Reynolds et al. 2017).

Interestingly, bacteria such as *Mycobacterium tuberculosis* have been predominantly reported from Nepalese elephants at and around various national parks of the country (Mikota et al. 2015; Paudel et al. 2014, 2019; Thapa et al. 2021). Thus, in this context, a concomitant infection caused by *M. tuberculosis* and GI parasites among captive and wild elephant populations in the study area can be a serious concern for their survival, suitable growth and welfare in Nepal. Therefore, a study involving the One Health approach, such as microbes from elephants, surrounding environmental samples, wild and domestic fauna and humans, will help define zoonosis and spillover of intestinal parasites in the national park areas.

## 5 Conclusion and Recommendations

This study first elaborated on the vast diversity and prevalence of intestinal protozoan and helminth parasites in the dung samples of Asian elephants outside the national park areas in central Nepal. This diversification might be due to overlapping niches with domestic and wild animals and humans, irregular medication and probable stressors. It can enhance the zoonotic potentialities, mainly due to widespread dung in the human inhabitant areas. Even though strategic planning and conservation efforts have been made for the healthy co‐existence of wild elephants and humans in the study area, little attention has been given to conservation and the welfare of captive elephants, considering their health threats, particularly GI parasitism. Therefore, the current results can help to sensitize government bodies, local organizations and conservationists regarding the existence of disease threats in the wild elephant population and facilitate them to design current and future preventive strategies. This information is essential to ensure the sustainable conservation and welfare of the endangered elephant population in Nepal. Further, a molecular study on shared parasites needs to be conducted among elephants, other domestic and wild fauna, humans and nearby environments (soil, water, and vegetation), mainly focusing on the One Health approach.

## Author Contributions

**Roshan Babu Adhikari**: Conceptualization (lead); methodology (lead); writing – original draft preparation (lead); formal analysis (lead); data curation (lead); field and laboratory investigation (lead); software (supporting); resources (supporting); visualization (supporting), writing – review and editing (equal).**Madhuri Adhikari Dhakal**: Formal analysis (supporting), writing – original draft (supporting). **Purna Bahadur Ale**: Software (supporting); writing – review and editing (supporting). **Ganga Ram Regmi**: Formal analysis (supporting), writing – review and editing (supporting). **Tirth Raj Ghimire**: Conceptualization (supporting), methodology (supporting), data curation (supporting), formal analysis (supporting), project administration (lead), software (lead), resources (lead), validation (lead), visualization (lead), writing – review and editing (equal)

## Ethics Statement

The required permission for research and collection of the faecal samples was issued by the Government of Nepal, Department of Forest and Soil Conservation (Permission No. 159/077/078), Division Forest Office, Bharatpur, Chitwan, Nepal (Permission No. 852/ (077/078) and Ratnanagar Municipality and Municipality Livestock and Veterinary Service, Ratnanagar Municipality, Chitwan, Nepal (Permission No. 1281/077/078). The authors declare that the study was conducted on dung samples defecated on the soil and that the faecal samples were from naturally infected captive Asian elephants. No experimental infection was established during this research work. None of the animals were touched, harmed or disturbed.

## Conflicts of Interest

The authors declare no conflicts of interest.

### Peer Review

The peer review history for this article is available at https://www.webofscience.com/api/gateway/wos/peer‐review/10.1002/vms3.70310.

## Data Availability

All data generated in the research have been submitted in this article.
